# Brain-targeted nanoreactors prevent the development of organophosphate-induced delayed neurological damage

**DOI:** 10.1186/s12951-023-02039-2

**Published:** 2023-08-07

**Authors:** Shuaijun Zou, Qianqian Wang, Qian He, Guoyan Liu, Juxingsi Song, Jie Li, Fan Wang, Yichao Huang, Yanan Hu, Dayuan Zhou, Yongfei Lv, Yuanjie Zhu, Beilei Wang, Liming Zhang

**Affiliations:** 1 Department of Marine Biomedicine and Polar Medicine, Naval Special Medical Centre, Naval Medical University, Shanghai, 200433 China; 2 The Third Affiliated Hospital, Naval Medical University, Shanghai, 200433 China; 3 Department of Marine Biological Injury and Dermatology, Naval Special Medical Centre, Naval Medical University, Shanghai, 200052 China

**Keywords:** Organophosphates, Enzyme immobilization, Nanoreactors, Delayed neurological damage, Brain targeting

## Abstract

**Background:**

Organophosphate (OP)-induced delayed neurological damage is attributed to permanent neuropathological lesions caused by irreversible OP-neurocyte interactions, without potent brain-targeted etiological antidotes to date. The development of alternative therapies to achieve intracerebral OP detoxification is urgently needed.

**Methods:**

We designed a brain-targeted nanoreactor by integrating enzyme immobilization and biomimetic membrane camouflaging protocols with careful characterization, and then examined its blood–brain barrier (BBB) permeability both in vitro and in vivo. Subsequently, the oxidative stress parameters, neuroinflammatory factors, apoptotic proteins and histopathological changes were measured and neurobehavioral tests were performed.

**Results:**

The well-characterized nanoreactors exerted favourable BBB penetration capability both in vitro and in vivo, significantly inhibiting OP-induced intracerebral damage. At the cellular and tissue levels, nanoreactors obviously blocked oxidative stress, cellular apoptosis, inflammatory reactions and brain histopathological damage. Furthermore, nanoreactors radically prevented the occurrence of OP-induced delayed cognitive deficits and psychiatric abnormality.

**Conclusion:**

The nanoreactors significantly prevented the development of OP-induced delayed neurological damage, suggesting a potential brain-targeted etiological strategy to attenuate OP-related delayed neurological and neurobehavioral disorders.

**Graphical Abstract:**

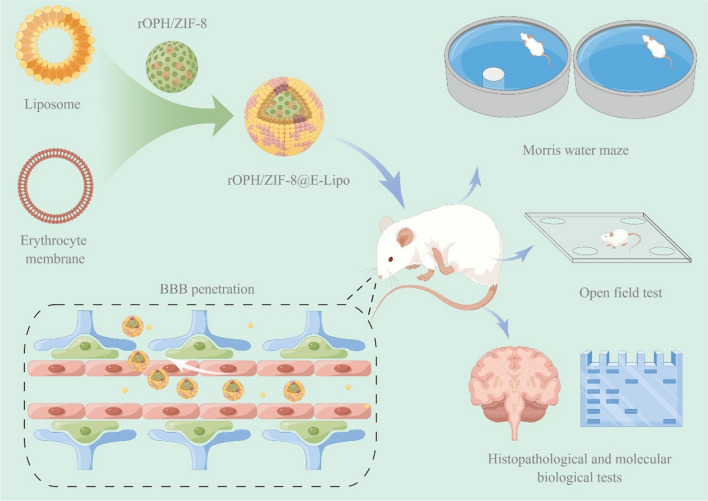

**Supplementary Information:**

The online version contains supplementary material available at 10.1186/s12951-023-02039-2.

## Introduction

Organophosphates (OPs) are potent neurotoxic chemicals that are widely used in industry and agriculture, leading to approximately 3 million poisonings and 300,000 deaths per year [1]. The toxicity of these compounds is associated with irreversible inhibition of acetylcholinesterase (AChE), which is responsible for terminating the action of the neurotransmitter acetylcholine (ACh) at cholinergic synapses. This process is particularly important to maintain essential functions of the central nervous system (CNS). Since most OPs are lipophilic compounds that easily penetrate the blood–brain barrier (BBB) and inactivate AChE, leading to irreversible neurocyte damage and even death [2–6], the survivors of OP poisoning may suffer from unacceptable neurodegenerative diseases, including cognitive deficits, depression and anxiety [7–11]. Long-term neurological and neurobehavioral impairments may be associated with permanent neuropathological lesions in different brain regions, including the cortex, hippocampus, thalamus and amygdala [12–17]. Therefore, the design of a brain-targeted drug delivery system to transport available therapeutic agents into the brain is currently the most promising strategy to alleviate the CNS toxicity of OPs.

At present, muscarinic receptor antagonists (*e.g.*, atropine) and cholinesterase reactivators (*e.g.*, oximes) are the most widely used clinical agents for the treatment of neurotoxic poisoning [18]. These classical medical countermeasures mitigate acute toxicity effects but have limited effects on OP-induced irreversible brain damage due to their poor penetration across the BBB. Thus, there have been active attempts to improve brain-targeted nano delivery systems loaded with conventional drugs to prevent CNS damage by OPs [19, 20]. To date, a variety of nanoparticles (NPs) with unique physicochemical properties have been tailored as brain‐targeted nano delivery systems, especially when functionalized with specific targeting ligands. These ligand‐functionalized NPs have been shown to actively recognize specific receptors on the BBB for brain-targeted drug delivery [21, 22]. For example, Wang [23] prepared a detoxification system consisting of mesoporous silica nanoparticles (MSNs) modified with a brain-targeted protein layer and loaded with the AChE reactivator (HI-6), which could effectively treat OP-induced brain poisoning. Quan [24] reported a brain-targeted detoxification system consisting of liposomes modified with transferrin receptor (TfR) aptamer and loaded with obidoxime for the treatment of OP poisoning. However, these preparations are essentially symptomatic rather than etiological treatments, thus showing deficiency of neuroprotection.

Ideally, strategies against OP poisoning should prevent casualties, alleviate symptoms, and minimize the development of incapacitation and long-term effects in poisoned persons. However, after more than 70 years of research, the pharmacological approaches used for the pre- and postexposure treatment of OP poisoning remain inadequate. Over the last three decades, alternative approaches have been explored, among which bioscavengers are the most promising alternative, as they neutralize OPs in the bloodstream before OPs reach their physiological targets, addressing OP poisoning etiologically. The first-generation bioscavengers refer to stoichiometric bioscavengers, which rely on huge doses for detoxification [25]. The second-generation bioscavengers are catalytic ones that greatly improve detoxification efficiency with a high turnover [26]. However, free bioscavengers tend to be quickly cleared due to their short circulation time and low plasma stability [27].

**Scheme 1:**
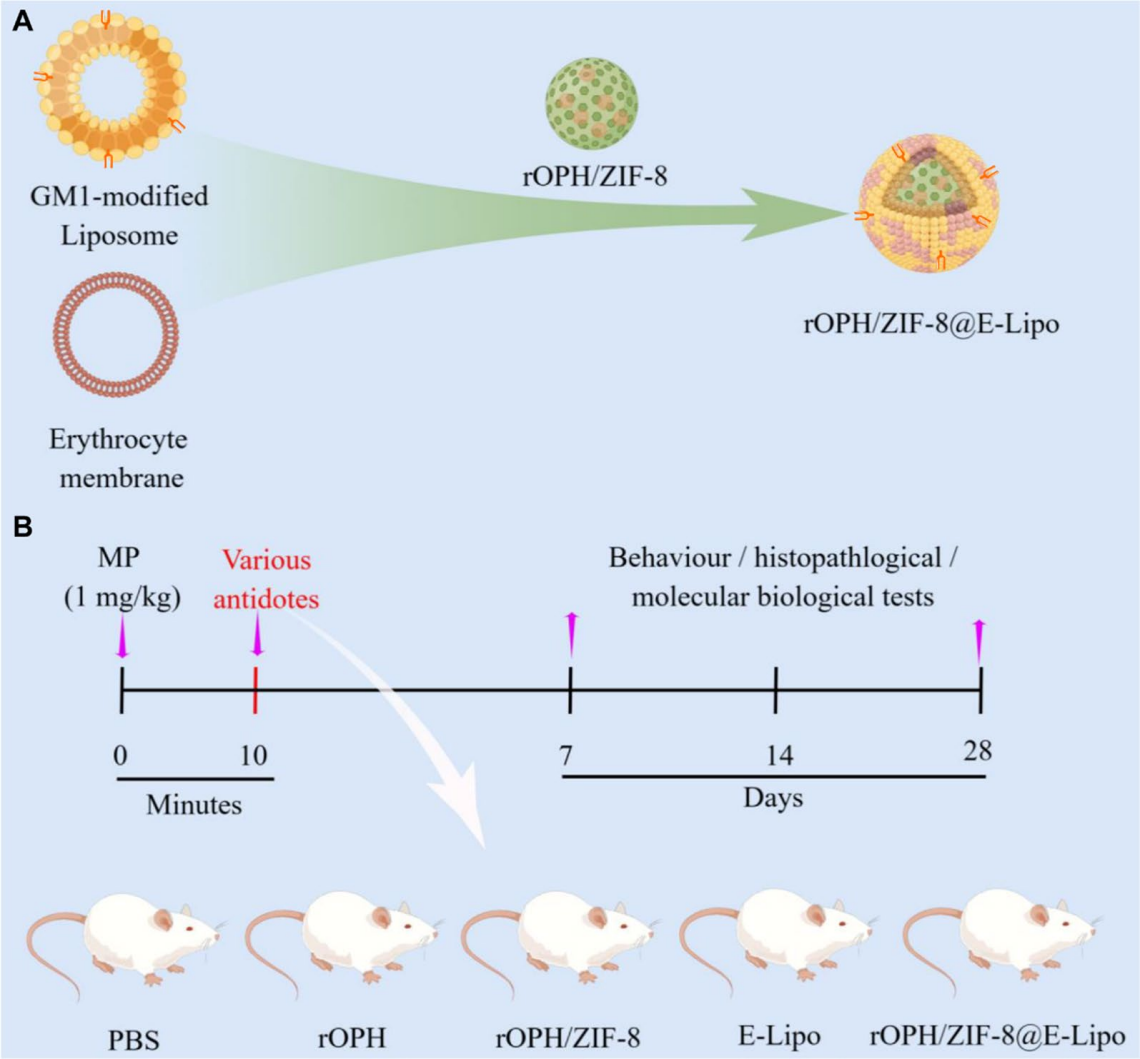
Paradigm for preparation and neuroprotective evaluation of nanoreactors. **A** The design and preparation of rOPH/ZIF-8@E-Lipo. **B** After treatment with various antidotes (PBS, rOPH, rOPH/ZIF-8, E-Lipo or rOPH/ZIF-8@E-Lipo), the poisoned rats underwent behavioural, histopathological, or molecular biological tests at predetermined time points

Herein, we developed a dual-modal nanoreactor by integrating enzyme immobilization and erythrocyte-liposome hybrid membrane (E-Lipo) camouflage techniques based on a stoichiometric bioscavenger (RBC-membrane-anchored AChE) and a catalytic bioscavenger (recombinant organophosphorus hydrolase, rOPH), in order to improve the detoxification efficiency and circulating stability of catalytic bioscavengers. Furthermore, we employed monosialoganglioside (GM1) to modify E-Lipo for BBB penetration, which is widely present on the surface of the BBB-constructed neuronal cells for mutual recognition and has been verified to assist nanocarriers in overcoming the BBB [28–30]. After that, we studied the physiochemical characterization of the nanoreactors. Then, we evaluated the BBB permeability of the nanoreactors via in vitro models, in vivo imaging and an intrathecal intoxication model. Finally, we further explored their protective effects against OP-induced delayed neuronal damage by examining cellular and tissue parameters, neuropathological lesions and neuropsychiatric changes (Scheme 1).

## Results

### Characterization of nanoreactors

**Fig. 1:**
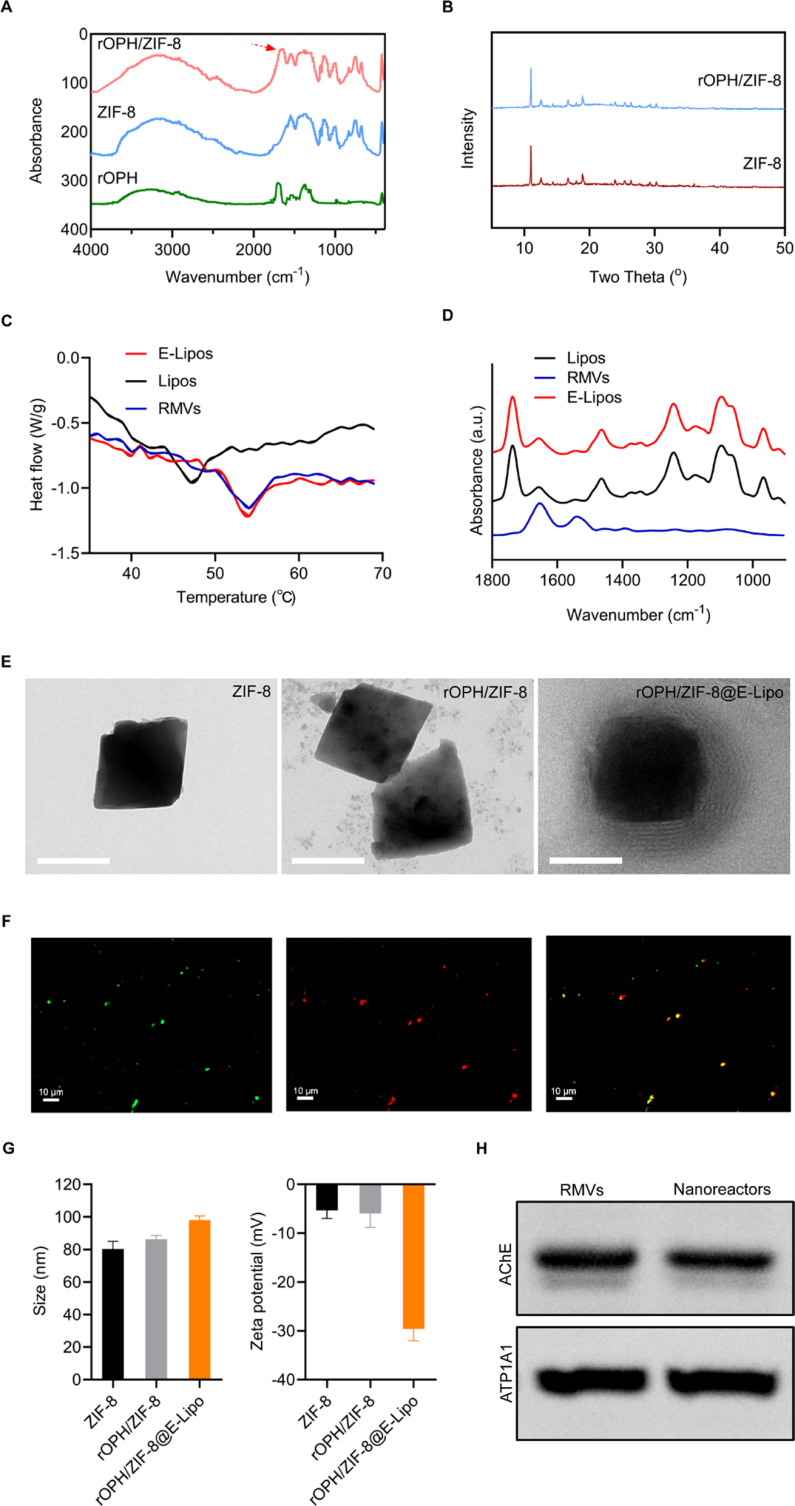
Characterization of nanoreactors. **A** FTIR spectra of the assembly of rOPH in ZIF-8 nanoparticles. **B** XRD patterns of ZIF-8 and rOPH/ZIF-8. **C** DSC measurement of liposomes (Lipos), red blood cell membrane vesicles (RMVs) and E-Lipos on the change in the Tm. **D** FT-IR spectra of Lipos, RMVs, and E-Lipos. **E** Representative TEM images of ZIF-8, rOPH/ZIF-8 and rOPH/ZIF-8@E-Lipo. **F** Confocal microscopy image of ZIF-8 nanoparticles camouflaged with fluorescence-labeled E-Lipo (green: rOPH/ZIF-8, red: E-Lipo). Scale bar: 10 μm. **G** Diameter and zeta potential of ZIF-8, rOPH/ZIF-8 and rOPH/ZIF-8@E-Lipo. **H** Western blotting of AChE on RMVs and rOPH/ZIF-8@E-Lipo. Data are shown as the means ± standard error (SE). n = 3

The physicochemical characterization of nanoreactors was sequentially performed during preparation. First, successful incorporation of rOPH in ZIF-8 nanoparticles is evidenced by the presence of characteristic protein absorption peaks at approximately 1667 cm^−1^ (C=O) in FTIR spectroscopy (Fig. 1A), without altering the crystal structure of ZIF-8 in the XRD assay (Fig. 1B). Furthermore, the rOPH loading efficiency is as high as ∼ 90% and the encapsulation efficiency is ∼ 10.1%.

To verify the fusion of the erythrocyte membrane and lipids, a DSC assay revealed that the transition temperature (Tm) of liposomes increased from 47.1 to 54.1 °C after fusion with the erythrocyte membrane, which was similar to that of the erythrocyte membrane (Fig. 1C). In addition, FT-IR spectroscopy revealed similar typical protein absorption bands between the erythrocyte membrane and E-Lipo hybrid membrane, indicating successful incorporation of the erythrocyte membrane into lipids (Fig. 1D).

After preparation of rOPH/ZIF-8@E-Lipo, TEM showed that the E-Lipo hybrid membrane could completely cover the ZIF-8 nanoparticles and form a distinctive “core–shell” structure compared to bare ZIF-8 nanoparticles (Fig. 1E). In addition, the confocal microscopy image showed an apparent fluorescence merge between rOPH/ZIF-8 nanoparticles and E-Lipo membranes (Fig. 1F). DLS examination further revealed that the diameter of rOPH/ZIF-8 nanoparticles increased from 86.3 ± 1.3 to 98.0 ± 1.5 nm after E-Lipo hybrid membrane covering (Fig. 1G, left). The zeta potential of rOPH/ZIF-8 nanoparticles decreased from − 6.0 ± 1.7 to − 29.6 ± 1.4 mV (Fig. 1G, right), where the latter one is approximate to the zeta potential of E-Lipo hybrid membrane.

Moreover, western blot analysis validated that the nanoreactors retained sufficient key membrane protein AChE similar to that of the RBC membrane, which can assist nanoreactors in MP detoxification (Fig. 1H).

Finally, given that nanoreactors play a role in the CNS, it is vital to examine their biocompatibility and safety in vivo. No significant changes in the routine blood examination and histopathologic examination were observed between the nanoreactor-treated and control groups (Additional file 1: Fig. S1).

### BBB permeability of nanoreactors

**Fig. 2:**
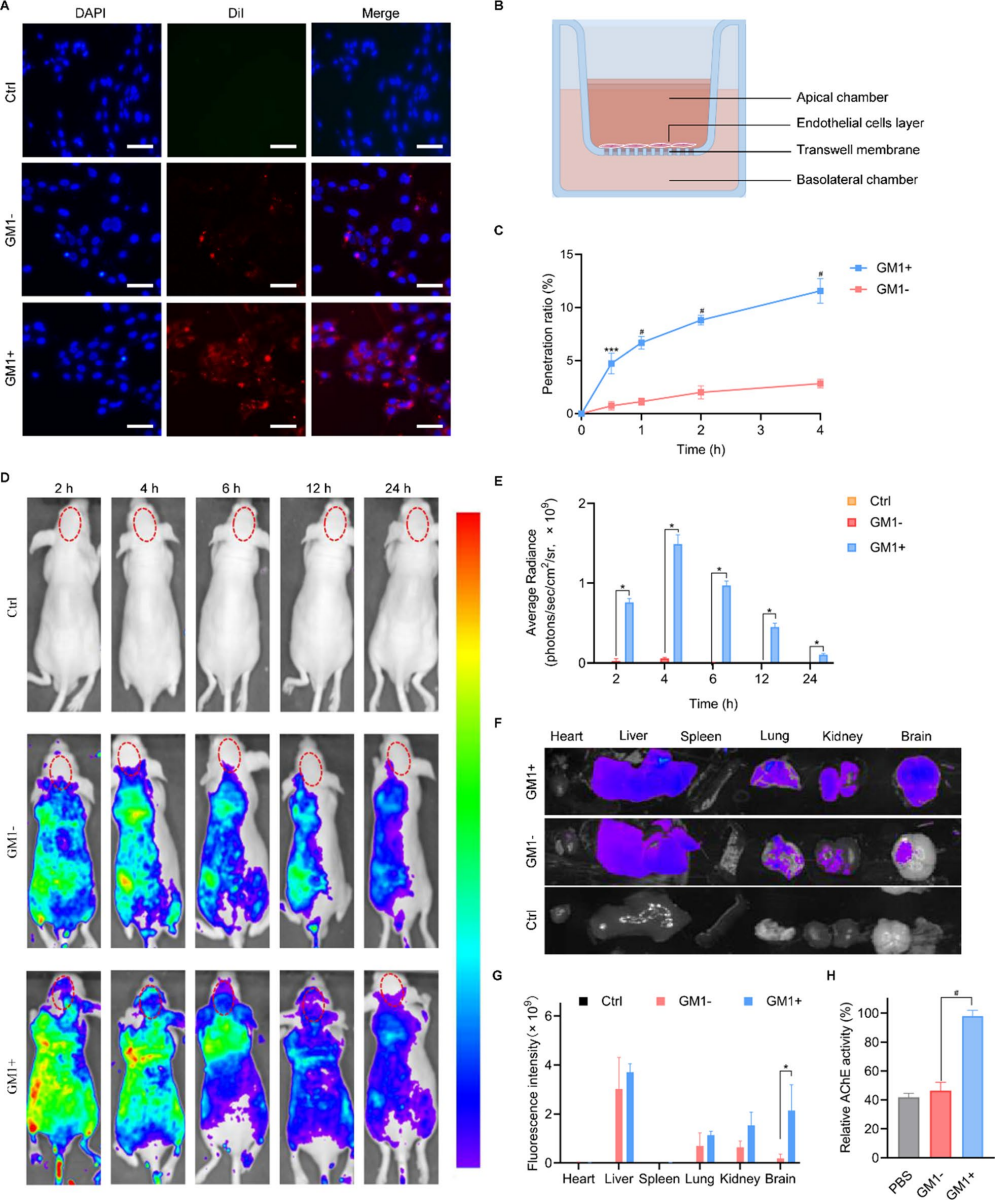
BBB penetration and intracerebral detoxification assays. **A** Representative fluorescence images of the uptake of nanoreactors with or without GM1 modification in bEnd.3 cells after incubation for 4 h, Scale bar = 50 μm. **B** Schematic representation of the BBB model penetration test. **C** Permeability of nanoreactors through the BBB model over a period of 4 h. **D** Fluorescence images of mice taken at certain time points (2, 4, 6, 12, 24 h). **E** Quantification of the fluorescence intensity of the circled zones in (**D**). **F** Representative fluorescence images and **G** quantification of fluorescence intensity of major organs at 4 h. **H** Cholinesterase activity of sublethal MP-challenged mice with different treatments. Data are shown as the means ± SE. n = 6. ^*^P < 0.05, ^***^P < 0.001, ^#^*P* < 0.0001. (GM1+ refers to GM1-modified nanoreactors, and GM1- refers to nanoreactors without GM1 modification)

We first prepared two types of DiI-labeled nanoreactors with or without GM1 modification. As shown in Fig. 2A, the uptake of 15 mol% GM1-modified rOPH/ZIF-8@E-Lipo (rOPH/ZIF-8@E-Lipo^GM1+^) by endothelial cells at 4 h was significantly higher than that of the nanoreactors without GM1 modification (rOPH/ZIF-8@E-Lipo^GM1−^). Furthermore, at different predetermined times, the permeability across the BBB model of rOPH/ZIF-8@E-Lipo^GM1+^ was higher than that of rOPH/ZIF-8@E-Lipo^GM1−^. The penetration ratio of rOPH/ZIF-8@E-Lipo^GM1+^ was 11.57 ± 1.16% at 4 h, while only 2.83 ± 0.42% of rOPH/ZIF-8@E-Lipo^GM1−^ passed through the BBB model (Fig. 2B, C). The above results demonstrate that GM1 modification potentially assists nanoreactors in penetrating the BBB.

The biodistribution of nanoreactors was assessed to detect their BBB penetration ability in vivo. As shown in Fig. 2D, E, obvious fluorescence signals were visualized in the brain zones of the mice treated with GM1-modified nanoreactors with a peak at 4 h and duration to 24 h, while the fluorescence intensity was weaker in the brain zones of mice treated with non-GM1-modified nanoreactors, demonstrating that GM1 is potent in assisting nanoreactors in penetrating the BBB. For ex vivo imaging of perfused organs at 4 h, weaker fluorescence intensity was detected in the brain of the rOPH/ZIF-8@E-Lipo^GM1−^ group than in the rOPH/ZIF-8@E-Lipo^GM1+^ group, with similar fluorescence distribution in other organs of the two groups, further validating the intracerebral distribution of nanoreactors (Fig. 2F, G).

To further demonstrate the BBB penetration of nanoreactors, intracerebral detoxification assays were performed. When mice were intrathecally poisoned by sublethal MP (0.525 mg/kg, 0.75× LD_50_, Additional file 1: Fig. S2), GM1-modified nanoreactors significantly inhibited the sublethal MP-induced decrease in intracerebral cholinesterase activity, while nanoreactors without GM1 modification did not exert protective effects (Fig. 2H). The results indicated that GM1 assisted nanoreactors in penetrating the BBB for MP detoxification in the CNS.

### Inhibition of neurocyte oxidative stress by nanoreactors

**Fig. 3:**
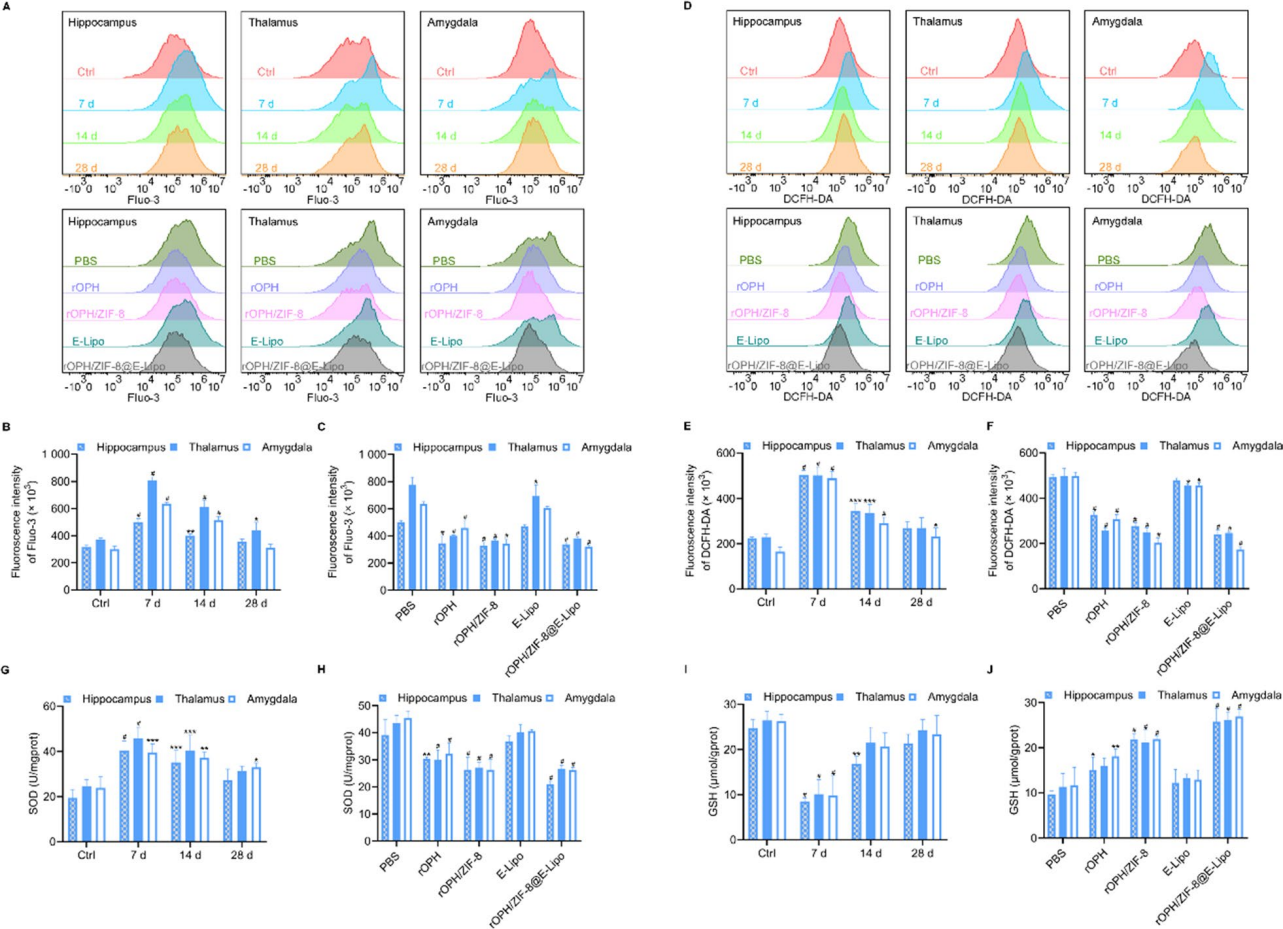
Oxidative stress assay in rat brains. **A** Histograms of Fluo-3 AM probes in different brain regions of poisoned rats with or without interventions. **B** Quantification of the fluorescence intensity of the upper images in (**A**). **C** Quantification of the fluorescence intensity of the lower images in (**A**). **D** Histograms of DCFH-DA probes in different brain regions of poisoned rats with or without interventions. **E** Quantification of the fluorescence intensity of the upper images in (**D**). **F** Quantification of the fluorescence intensity of the lower images in (**D**). **G** SOD activity of control rats and MP-challenged rats at 7 d, 14 d, and 28 d. **H** SOD activity of each group of rats with various therapeutic antidotes. **I** Reduced GSH concentration of control rats and MP-challenged rats at 7 d, 14 d, and 28 d. **J** Reduced GSH concentration of each group of rats with various therapeutic antidotes. Data are shown as the means ± SE. n = 3. ^*^P < 0.05, ^**^P < 0.01, ^***^P < 0.001, ^#^*P* < 0.0001

Strong evidence suggests that excessive amounts of Ca^2+^ are responsible for ROS production, which further contributes to oxidative stress to trigger neurotoxicity [31]. Therefore, we first examined levels of oxidative parameters (Ca^2+^ and ROS) and endogenous antioxidants (SOD and reduced GSH) in the hippocampus (including CA1, CA3 and DG subregions), thalamus, and amygdala using Fluo-3 AM and DCFH-DA fluorescence probes, respectively. Intracellular fluorescence indicating Ca^2+^ influx and ROS production was apparently detected in the poisoned rats at 7 d after MP intoxication but was negligibly obvious in the 14 d and 28 d groups (Fig. 3A, B, D, E). We thus studied the protective effects of nanoreactors at 7 d after MP poisoning. rOPH-containing antidotes, especially nanoreactors, exhibited better inhibitory effects against Ca^2+^ influx and ROS production than the others without rOPH (Fig. 3A, D, E, F). We found that nanoreactors exerted the best effect on SOD and reduced GSH to inhibit the imbalance of these antioxidants among all groups (Fig. 3G–J). All these results indicated that nanoreactors can significantly inhibit oxidative stress in brain tissues.

### Nanoreactors blocked the activation of intracellular apoptotic factors

**Fig. 4:**
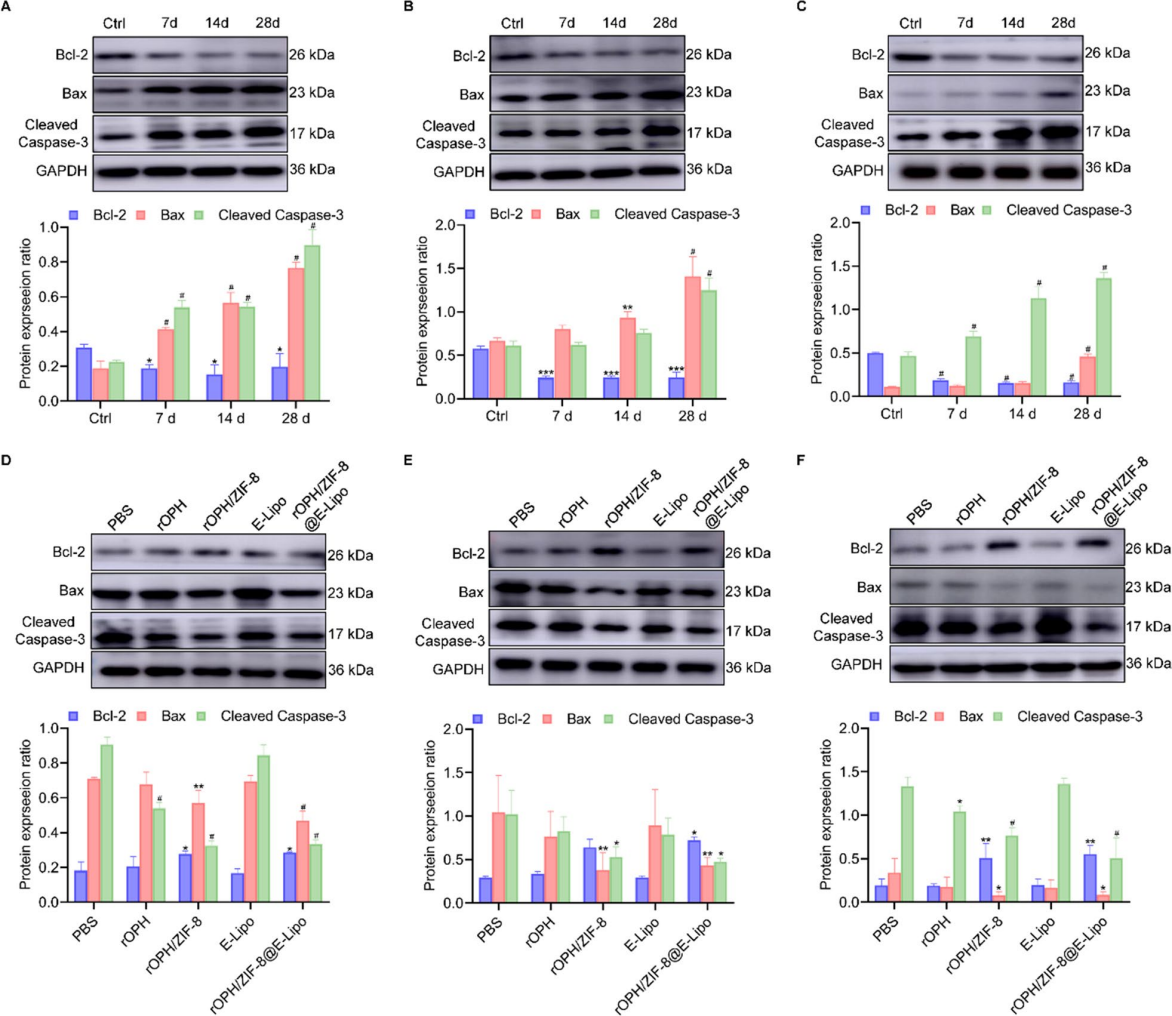
Nanoreactors inhibited MP-induced activation of apoptotic factors. Expression of Bcl-2, Bax and caspase-3 in the Ctrl group and MP-challenged rats at 7 d, 14 d, and 28 d in the **A** hippocampus, **B** amygdala and **C** thalamus. Expression of Bcl-2, Bax and caspase-3 28 d after MP intoxication and treatment with various antidotes in the **D** hippocampus, **E** amygdala and **F** thalamus. Data are shown as the means ± SE. n = 3. ^*^P < 0.05, ^**^P < 0.01, ^***^P < 0.001, ^#^*P* < 0.0001

Oxidative stress-induced mitochondrial damage can potentially activate caspases which are the primary markers of neuronal apoptosis or necrosis [32–34]. Therefore, we further examined the expression levels of several main apoptosis executors, including Bcl-2, Bax and caspase-3. Compared to the control group, MP (1 mg/kg) significantly altered the Bcl-2/Bax ratio and caspase-3 content from 7 d after MP intoxication in some brain regions and progressively aggravated apoptosis at 14 d and 28 d in the hippocampus, thalamus and amygdala (Fig. 4A–C). We thus treated poisoned rats with various antidotes and examined them at 28 d. It was found that nanoreactors also exerted the best inhibitory effects on imbalanced Bcl-2/Bax expression and abnormal activation of caspase-3 in these brain zones among all the treatment groups (Fig. 4D–F). All these results indicated that nanoreactors can significantly prevent MP-induced delayed apoptotic injuries.

### Inhibition of MP-induced neuroinflammation by nanoreactors

**Fig. 5:**
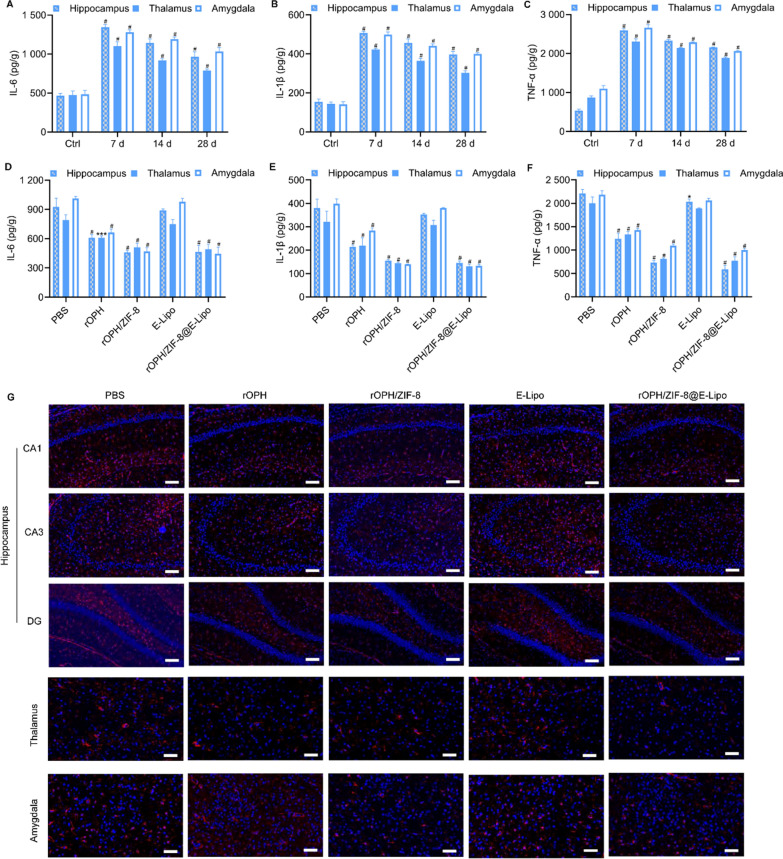
Neuroinflammation examination in rat brains. **A** IL-6, **B** IL-1β and **C** TNF-α levels in the hippocampus, thalamus and amygdala of control rats and MP-challenged rats at 7 d, 14 d, and 28 d. **D** IL-6, **E** IL-1β and **F** TNF-α levels in the hippocampus, thalamus and amygdala at 28 d after MP poisoning and treatment with various therapeutic antidotes. **G** GFAP-positive astrocytes were observed under a fluorescence microscope with immunofluorescent staining. Scale bar = 20 μm. Data are shown as the means ± SE. n = 3. ^*^P < 0.05, ^***^P < 0.001, ^#^*P* < 0.0001

Neuroinflammation can be evoked by apoptosis and cell death as a late consequence, leading to neurologic or neuropsychiatric conditions [35]. We thus examined multiple neuroinflammatory factors, including proinflammatory cytokines (IL-6, IL-1β, TNF-α) and the astrocyte activation marker GFAP [36]. After MP intoxication, overexpression of IL-6, IL-1β and TNF-α was detected at 7 d, 14 d and 28 d in the hippocampus, thalamus, and amygdala, indicating a neuroinflammatory response in these regions (Fig. 5A–C). Accordingly, GFAP levels were also elevated and positive cells were progressively aggregated until 28 d after poisoning, indicating the presence of activated astrocytes in those brain zones (Additional file 1: Fig. S3). We thus adopted various antidotes to intervene and examine the rats at 28 d after poisoning. The results demonstrated that proinflammatory cytokines (IL-6, IL-1β, TNF-α) were inhibited to various degrees. Therein, rOPH-containing antidotes significantly inhibited the activation of proinflammatory cytokines, and the nanoreactors exerted the best protective effect (Fig. 5D–F). Similarly, nanoreactors completely prevented GFAP upregulation and astrocyte activation and provided the best protection among all the treatment groups, indicating a significant inhibition of MP-induced neuroinflammation by nanoreactors (Fig. 5G).

### Protective effects of nanoreactors against delayed neuropathological changes

**Fig. 6:**
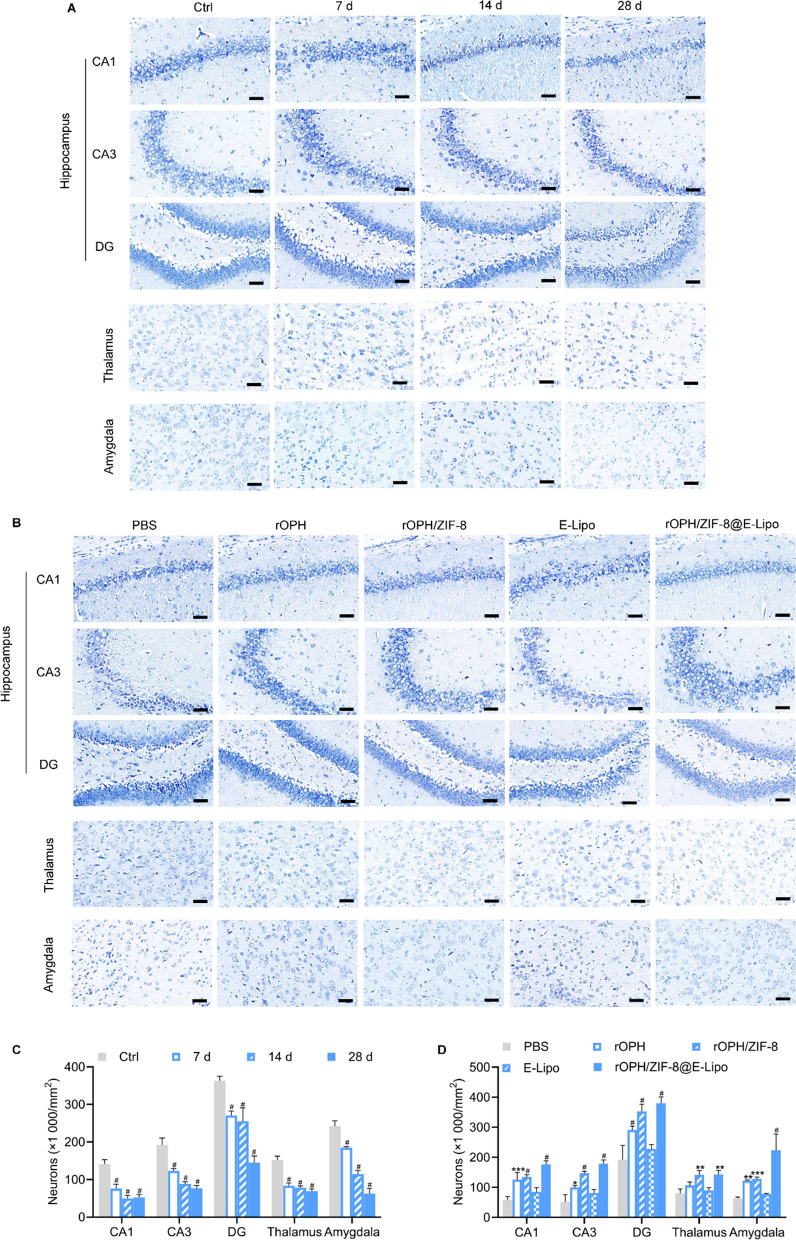
Histopathology with Nissl staining. **A** Neurocytes in the hippocampus, thalamus and amygdala of the control group and MP-challenged rats at 7 d, 14 d, and 28 d. **B** Neurocytes in the hippocampus, thalamus and amygdala of MP-challenged rats treated with various antidotes at 28 d. Scale bar = 20 μm. **C** Quantification of neurocytes in (**A**). **D** Quantification of neurocytes in (**B**). Data are shown as the means ± SE. n = 3. ^*^P < 0.05, ^**^P < 0.01, ^***^P < 0.001, ^#^*P* < 0.0001

The pathological effects of MP on neurocytes in the hippocampus (including the CA1, CA3 and DG subregions), thalamus, and amygdala were examined via Nissl staining. As a neural characteristic, the number of Nissl bodies reflects the survival state of neurocytes. In the control group, normal neuronal cells were observed with oval rounded basophilic nuclei. After MP (1 mg/kg) contamination, neurocytes showed shrinkage with condensed and darkly stained nuclei and sparse Nissl bodies from 7 d after MP intoxication and even a significant numerical loss at 28 d, indicating apparent neuronal damage (Fig. 6A, C). After therapeutic intervention with various antidotes, neuronal damage at 28 d was alleviated to various degrees. Nanoreactors exerted the best protective effect among all treatment groups (Fig. 6B, D), demonstrating excellent therapeutic efficacy against MP-induced delayed neuronal pathological damage.

### Nanoreactors prevented MP-induced delayed neurobehavioural disorders

**Fig. 7:**
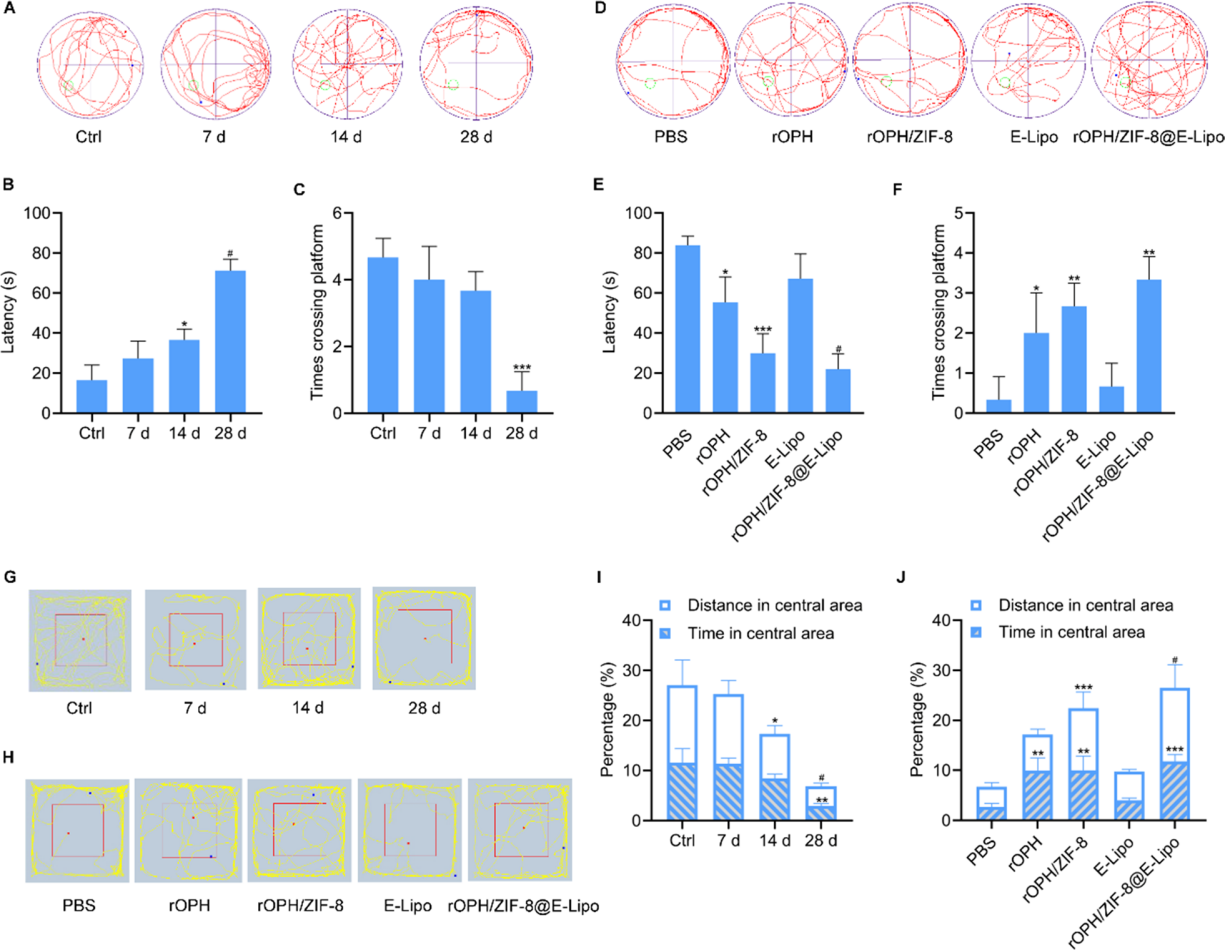
Preventative effects of nanoreactors on neurobehavioral disorders of rats. **A** Representative images of routes of MP-challenged rats in the water maze test. **B**, **C** Quantification of the number of platform crossings and the latency to first entry on different days. **D** Representative images of routes of MP-challenged rats in the water maze test with various interventions. **E**, **F** Quantification of platform crossings and the latency to first entry of different treatment groups. **G** Representative images of routes of MP-challenged rats in the open field test. **H** Representative images of routes of MP-challenged rats in the open field test with various interventions. **I** Quantification of distance travelled and time spent in the central area of rats on different days. **J** Quantification of distance and time in the central area of rats treated with different therapeutic antidotes. Data are shown as the means ± SE. n = 3. ^*^P < 0.05, ^**^P < 0.01, ^***^P < 0.001, ^#^*P* < 0.0001

To examine the preventative effects of nanoreactors on MP-induced cognitive deficits, rats were subjected to behavioural evaluation with the Morris water maze test. For poisoned rats, the latency to find the platform during training negligibly changed in the 7 d and 14 d groups but significantly increased in the 28 d group (Additional file 1: Fig. S4). During the exploration stage after platform removal, the number of platform crossings decreased at 14 and 28 d, but the latency time only increased at 28 d (Fig. 7A–C), further demonstrating the impairment of learning and memory abilities 28 d after MP intoxication. We thus examined the preventive effects of different antidotes at 28 d after poisoning. For MP detoxification, rOPH/ZIF-8@E-Lipo provided the best prevention of MP-induced cognitive disorders compared to the other groups, which not only shortened the time to find the platform during training (Additional file 1: Fig. S5) but also increased the number of platform crossings and decreased the latency time to different degrees during the exploration stage (Fig. 7D–F). All the results indicated that nanoreactors can apparently improve MP-induced delayed cognitive deficits.

For the open field test, the distance travelled in the central area of MP-challenged rats decreased in the 14 d group and apparently declined in the 28 d group, and the time spent in the central area only declined in the 28 d group (Fig. 7G, I), manifesting a significant symptom of anxiety and depression at 28 d after MP poisoning. After therapeutic intervention, poisoned rats showed enhanced activity in the central area to varying degrees at 28 d. Compared to the other groups, the nanoreactors prevented the poisoned rats from suffering from anxious or depressed symptoms (Fig. 7H, J), suggesting that the nanoreactors can significantly prevent the development of neuropsychiatric disorders caused by MP.

## Discussion

Various epidemiological studies have demonstrated that a single acute exposure or repeated subclinical doses of OPs can result in delayed neuronal damage, which often continues for weeks to years after exposure, with neurological and neurobehavioral abnormalities [37, 38]. Traditional antidotes are insufficient to prevent the development of neuronal damage due to poor BBB permeability [39]. In addition, the existing brain-targeted antidotes are essentially symptomatic rather than etiological therapies, with deficiencies in neuronal protection [24, 40]. Therefore, it is urgent to design alternative brain-targeted etiological nano-antidotes. Herein, we developed a novel nanoreactor (rOPH/ZIF-8@E-Lipo), which not only efficiently penetrates the BBB, but also radically removes neurotoxic agents, thus significantly preventing MP-induced delayed neurological damage.

GM1 gangliosides consist of a hydrophilic sialic acid terminal sugar and a hydrophobic ceramide moiety, widely existing on the surface of neuronal cells. It plays neuroprotective and neurorestorative roles in neuronal injuries, and more importantly, it can overcome the BBB to treat neurological diseases [41]. Therefore, GM1-modified nanocarriers have been developed as drug delivery protocols targeting the CNS. For example, Mora *et al*. [42] found that GM1 modification makes liposomes good candidates for brain-targeted drug delivery systems. Zou *et al*. [30] designed GM1-modified nanomicelles that can penetrate the BBB and deliver doxorubicin to glioma. In general, the feasibly modified artificial liposomes are superior materials for linking GM1 and other targeting ligands. However, artificial liposomes entering the body lack sufficient circulatory stability and tend to be cleared by the immune system. To overcome this drawback, we constructed hybrid membranes by fusing liposomes to erythrocyte membrane, as the latter is highly stable in vivo with less immunogenicity [43]. In addition, erythrocyte membrane can also absorb and neutralize OPs via a stoichiometric bioscavenger (erythrocyte-membrane-anchored AChE) [44], thus enhancing detoxification efficacy of the nano-systems.

In the present study, GM1-modified nanoreactors were proved with satisfactory physiochemical characterization (Fig. 1), which can not only internalize into brain vascular endothelial cells and cross the BBB model in vitro but also apparently distribute into the brain in vivo. Additionally, we confirmed that peripherally used nanoreactors could penetrate the BBB and clear intrathecally injected. As a consequence, the nanoreactors significantly inhibited the decline in AChE activity in the brain, which may account for long-term and delayed neurological disorders (Fig. 2). Furthermore, our study proved that nanoreactors exerted satisfying biocompatibility and safety, which is crucial and fundamental for brain-targeted antidotes in vivo (Additional file 1: Fig. S1). To date, much progress has been made in the synthetic NP-based brain-targeted antidotes for OP poisoning. However, the existing antidotes mainly exert detoxification effects by reactivating AChE. For example, solid lipid nanoparticles (SLNs) were adopted to target the BBB for oxime delivery and prevent OP poisoning [45, 46]. Thiamine-modified pralidoxime chloride-loaded MIL-101-NH2 (Fe) nanoparticles were shown to be effective in crossing the BBB and restoring AChE activity in the brain of poisoned mice [40]. Unlike existing brain-targeted nano-antidotes, our nanoreactors (rOPH/ZIF-8@E-Lipo) act as a nanoscavenger by competitively removing and catalytically hydrolysing OPs. This is the first report on the use of bioscavenger-loaded nanomaterials for intracerebral detoxification of OPs, exerting etiological detoxification effects against neuronal cell and tissue damage rather than merely reactivating AChE. In addition to AChE inhibition (Fig. 2H), a noncholinergic mechanism may play an important role in OP-related delayed neuronal deficits [5, 47]. It was reported that oxidative stress is a common noncholinergic mechanism of OP neurotoxicity, among which Ca^2+^ influx is responsible for ROS formation that further contributes to mitochondrial injury-related apoptosis [48–50]. Apoptosis in turn accounts for the cascade of proinflammatory cytokines (IL-6, IL-1β and TNF-α) and neuroinflammation, leading to persistent neuronal damage [33]. In our study, we confirmed that OP induced oxidative stress at an early stage (7 d after poisoning) and progressively exacerbated apoptosis, neuroinflammation and histopathological damage until 28 d (Figs. 3, 4, 5, 6). Overall, we speculated that nanoreactors can penetrate the BBB to protect neurocytes by radically eliminating nerve agents and blocking OP-neurocyte interactions, thus inhibiting oxidative stress, apoptosis and neuroinflammation and further preventing delayed neurological and neurobehavioral disorders (Fig. 7).

## Conclusion

In this study, we developed a brain-targeted nanoreactor to etiologically prevent OP-induced delayed neurological damage. It can penetrate the BBB to eliminate intracerebral OPs, thus inhibiting oxidative stress, neuroinflammation and neuronal apoptosis of neurocytes. This study represents the first report of brain-targeted nanoreactors for OP-induced delayed intracerebral disorders.

## Methods

### Animals and reagents

All animal experiments were performed according to the Guide for the Care and Use of Laboratory Animals and were approved by the Medical Ethics Committee of Naval Medical University.

4ʹ,6-Diamidino-2-phenylindole (DAPI) and RIPA lysis buffer were purchased from Beyotime Biotechnology (Shanghai, China). Methyl paraoxon (MP), DCFH-DA and Fluo-3 AM fluorescence probes, IL-6, IL-1β, and TNF-α ELISA kits and goat serum were purchased from Thermo Fisher Scientific (MA, USA). Antibodies against glial fibrillary acidic protein (GFAP), Bcl-2, Bax, Caspase-3, and Cy3 or HRP-labelled IgG were purchased from Cell Signalling Technology (Shanghai, China). 1,2-Dipalmitoyl-sn-glycero-3-phospho-(1ʹ-rac-glycerol) (DPPG), cholesterol, 1,2-distearoyl-sn-glycero-3-phosphore-thanolamine-n-[methoxy (polyethylene glycol)-2000] (DSPE-mPEG2000) and monosialoganglioside GM1 (ovine) were purchased from Avanti Polar Lipids (AL, USA). Zn(NO_3_)_2_·6H_2_O and 2-methylimidazole were purchased from Solarbio Science & Technology Co., Ltd. (Beijing, China). All other reagents were of analytical grade, commercially available and used as received.

### Preparation of nanoreactors

Briefly, 280 mg 2-methylimidazole and 20 mg rOPH (prepared in our laboratory) were solubilized in 36 mL deionized water (DI water) (solution A); 70 mg Zn(NO_3_)_2_⋅6H_2_O was dissolved in 4 mL DI water in parallel (solution B). Solutions A and B were mixed, sonicated, magnetically stirred and centrifuged to obtain rOPH/ZIF-8 nanoparticles. Then, DPPG, cholesterol, DSPE-mPEG2000 and GM1 (50:30:5:15, M/M) were dissolved in 10 mL of dichloromethane to a final concentration of 4.5 mg/mL and evaporated to form a thin film. After preparing the murine erythrocyte membranes, the lipid film was hydrated with 10 mL water containing erythrocyte membranes (equal to the amount of lipids) and the previously obtained rOPH/ZIF-8, with serial extrusion through 400 nm, 200 nm and 100 nm polycarbonate porous membranes to obtain nanoreactors (rOPH/ZIF-8@E-Lipo).

### Characterization of nanoreactors

First, to verify the incorporation of rOPH into ZIF-8 nanoparticles, Fourier transform infrared (FT-IR) spectra were recorded and scanned from 400 to 4000 cm^−1^ at a resolution of 4 cm^−1^. The crystal structure of ZIF-8 nanoparticles with or without rOPH loading was examined by *X*-ray diffraction (XRD). Moreover, the encapsulation and loading efficiency of rOPH were calculated as follows:

1 $$\mathrm{Encapsulation \,efficiency }\left(\mathrm{\%}\right)=\mathrm{ M}1/\mathrm{M }\times 100\mathrm{\%}$$

2 $$\mathrm{Loading \,efficiency }(\mathrm{\%}) =\mathrm{ M}1/\mathrm{M}2 \times 100\mathrm{\%}$$

where M1 is the amount of loaded rOPH, M2 is the total mass of nanoreactor, and M is the initial amount of rOPH. The amount of enzyme was calculated via an activity assay.

Next, the fusion of erythrocyte membranes and liposomes was verified using differential scanning calorimetry (DSC) and FT-IR spectra analyses. Briefly, lyophilized samples were placed in an alumina pan for DSC measurement under a nitrogen atmosphere, and the heating scan was performed from 35 to 70 °C at a rate of 5 °C/min. In a parallel experiment, lyophilized samples were pressed into pellets to detect the vibrational spectra from 400 to 4000 cm^−1^. A physical mixture of erythrocyte membrane, lipid membrane and rOPH/ZIF-8 nanoparticles was used as the control.

The hydrodynamic diameter, polydispersity index (PDI) and zeta potential of ZIF-8, rOPH/ZIF-8 and rOPH/ZIF-8@E-Lipo nanoparticles were measured using dynamic light scattering (DLS), and the structures of rOPH/ZIF-8 and rOPH/ZIF-8@E-Lipo were examined using transmission electron microscope (TEM) (Leica, Germany). Moreover, rOPH loaded in ZIF-8 nanoparticles was labelled with fluorescein isothiocyanate isomer and E-Lipo was labelled with Cy5 for confocal microscopy imaging.

Specific membrane protein detection, such as AChE on erythrocyte membranes and hybrid membranes, was performed using SDS-PAGE on a 10% Bis–Tris gel that was run at 120 V for 1 h, followed by a western blot assay.

### Biocompatibility of nanoreactors

Male ICR mice (20 ± 2 g) were intravenously injected with saline or nanoreactors (containing 10 mg/kg rOPH) every other day for 1 week (n = 6). Twenty-four hours after the last injection, whole blood or serum was collected for haematology and blood chemistry tests. Moreover, histopathological examination of major organs (heart, liver, spleen, lungs, kidneys and brain) was performed via haematoxylin–eosin (H&E) staining.

### Cellular uptake and BBB penetration in vitro

First, mouse brain endothelial (bEnd.3) cells were seeded into 6-well plates (1 × 10^5^ cells per well) and cultured for 24 h. The cells were then washed and treated with GM1- or non-GM1-modified nanoreactors (equivalent to 0.1 mg/mL rOPH) for 4 h. Next, the cells were washed and fixed in 4% paraformaldehyde for 10 min, followed by DAPI staining. Finally, the intracellular fluorescence intensity was detected by fluorescence microscopy (Leica, Wetzlar, Germany).

In addition, a BBB penetration test was performed via an in vitro model. First, rat brain microvascular endothelial cells (BMECs) at a density of 1 × 10^5^ cells were seeded on fibronectin-coated Transwell plates (Corning, New York, USA) and cultured until the transendothelial electrical resistance reached 200 Ω cm^2^. Next, GM1- or non-GM1-modified nanoreactors were added to the apical chamber. Nanoreactors in the basolateral chamber were detected and calculated by enzyme activity assay at predetermined times (0.5, 1, 2, 4 h).

### In vivo imaging

FITC-labelled nanoreactors (containing 10 mg/kg rOPH) were intravenously administered to nude mice (n = 6), and nanoreactors without GM1 modification or saline were injected into the control groups. At predetermined time points (2, 4, 6, 12, 24 h), biodistribution was detected using an imaging system (Bio-Real Quick View 3000, Austria). The fluorescence intensity of nanoreactors in the brain region was calculated and quantified. Given that the fluorescence intensity reached a peak at 4 h after administration, we further assessed the biodistribution of nanoreactors in the brain and other major organs of each group using an imaging system at this time point.

### Intracerebral detoxification efficacy of nanoreactors

To further evaluate and determine the BBB permeability of nanoreactors, intoxication was directly performed via intrathecal injection while antidotes were intravenously administered via tail veins. Briefly, GM1-modified nanoreactors (equivalent to 1 mg/kg rOPH) were administered (*i.v.*) 4 h before MP (0.525 mg/kg, 0.75× LD_50_) intoxication with intrathecal injection, followed by recording of cholinesterase activity in brain tissues. Mice pretreated with nanoreactors without GM1 modification or PBS were used as the control (n = 3).

### Measurement of oxidative stress markers

To examine the time-effect relationship of oxidative stress induced by MP on neurocytes, each group of Wistar rats were subcutaneously poisoned by MP (1 mg/kg, 0.8× LD_50_) and fed for 7 d, 14 d or 28 d, and rats subcutaneously injected with PBS were used as the control (n = 3). To investigate the detoxification of nanoreactors against MP, PBS, rOPH, rOPH/ZIF-8, E-Lipo and rOPH/ZIF-8@E-Lipo were injected (*i.v.*) to Wistar rats (n = 3) 10 min after MP poisoning. After processing, brain tissues of each brain region were immediately isolated from the rats of each group to prepare a single-cell suspension. Briefly, fresh tissue was minced using tissue strainers, and a suspension was prepared in PBS with centrifugation at 1200 × *g* for 10 min at 4 °C. Subsequently, for Ca^2+^ detection, isolated neurocytes were loaded with 1 μM Fluo-3 AM fluorescent probes at 37 ℃ for 30 min. After rinsing with PBS, the cell suspension was measured by flow cytometry. Similarly, to evaluate the levels of reactive oxygen species (ROS), isolated neurocytes from different regions were incubated with 10 μM DCFH-DA fluorescent dye at 37 ℃ for 30 min. After rinsing with PBS, ROS in the cell suspension were measured via a flow cytometer. Furthermore, the superoxide dismutase (SOD) activity and reduced glutathione (GSH) level of different brain regions were examined according to the instructions of appropriate assay kits.

### Apoptotic factor detection

We further explored the effects of MP on apoptosis-related factors with or without nanoreactor intervention. Wistar rats were divided into 4 groups (n = 3) for OP-induced apoptosis detection and 5 groups (n = 3) for detoxification assessment according to the protocols described above. After processing, brain tissues from different brain regions of rats were harvested, homogenized and centrifuged to obtain supernatants. The protein concentration was then determined, and the samples were preserved at − 20 ℃ for further assays. Twenty milligrams of protein from each sample were resolved by 10% SDS-PAGE and transferred to a polyvinylidene difluoride membrane. The membrane was blocked with 5% skim milk for 2 h and incubated with the appropriate primary antibody (1:500) at 4 °C overnight. Subsequently, the primary antibody was harvested, followed by washing and horseradish peroxidase-conjugated secondary antibody (1:1000) incubation for 1 h at room temperature. Finally, the membrane was rinsed with enhanced chemiluminescence solution for imaging.

### Assays of proinflammatory cytokines and astrocyte activation

The rats were divided into 4 groups (n = 3) for OP-induced proinflammatory cytokine cascade detection and 5 groups (n = 3) for detoxification assessment according to the protocols described above. Similarly, brain tissues from different brain regions were quickly harvested, homogenized and centrifuged to obtain supernatants. The protein concentration was then determined, and the samples were preserved at – 20 ℃ for subsequent examinations. Proinflammatory cytokines such as IL-6, IL-1β and TNF-α were detected by ELISA according to the instructions of the assay kits. Briefly, standard curves were first prepared in the supplied diluent with a range of 4000 to 125 pg/mL. Samples (100 μL per well) were added to 96-well plates and incubated for 120 min, with the plate washed 5 times. Then, the corresponding antibodies were added and incubated for 60 min. Next, the wells were washed, and streptavidin was added. Finally, the wells were washed again, and 3,3ʹ,5,5ʹ-tetramethylbenzidine solution was added for detection.

Next, GFAP immunofluorescence staining was also conducted to detect the activation of astrocytes. Briefly, brain tissue sections were rinsed in phosphate buffered saline (PBS) 3 times and then blocked in PBST containing 1.5% goat serum for 1 h. The sections were incubated with mouse monoclonal anti-GFAP antibody (1:500) overnight at 4 °C, washed with PBS 3 times and incubated with secondary antibody (1:500) for 2 h at room temperature. Subsequently, the nuclei were stained with DAPI for 5 min. The sections were then observed under a fluorescence microscope, and the number of GFAP-positive astrocytes in different sections was counted.

### Neuropathologic examination

Considering that the number of Nissl bodies reflects the survival state of neurocytes, we performed Nissl staining on brain tissue sections [32]. The rats were divided into 4 groups (n = 3) for OP-induced neuropathology detection and 5 groups (n = 3) for detoxification assessment according to the protocols described above. Briefly, the sections fixed in formalin and embedded in paraffin were deparaffinized in xylene and rehydrated in different grades of alcohol (100%, 90%, and 75%) 2 times for 1 min each, followed by a dip in DI water. Then, the sections were stained with 0.5% toluidine blue, and excess stain was removed by a quick rinse in DI water, followed by rinsing in differentiating solution. The sections were then dehydrated in 100% alcohol for 5 min 2 times. Neurocytes were evaluated in the cornu ammonis (CA)1, CA3 and dentate gyrus (DG) regions of the hippocampus, thalamus and amygdala under a microscope. We counted only well-rounded neurocytes with visible nucleoli in three separate nonadjacent sections from each animal using ImageJ software. The results were expressed as the mean value of neurocytes per square millimetre.

### Neurobehavioral tests in rats

The rats were divided into 4 groups (n = 3) for OP-induced neurobehavior abnormality detection and 5 groups (n = 3) for detoxification assessment according to the aforementioned protocols, followed by Morris water maze and open field tests.

The Morris water maze test was applied to measure the cognitive functioning of rats. Briefly, the rats challenged with MP or treated with various antidotes were trained to find the platform over 4 days after being placed in each quadrant. The time spent to locate the platform (escape latency) was recorded for each trial. After removal of the platform, the escape latency (s) and times crossing in the target quadrant were recorded using a tracking system on day 5.

For the open field test, an increased ratio in time and distance in the central part of the open field is considered to be an indication of anxiolytic behaviour. The open field is a square arena (45 cm × 45 cm × 35 cm) with an open top, dark walls and floor. The arena was subdivided into corner and centre zones. A camera was installed above the centre of the field. After the rat was placed in the corner of the open field, the movement and position of the rat were recorded. The time spent in the centre zone and the total distance moved during a 5 min test were analysed.

### Statistical analysis

Data are presented as the mean ± standard error (SE). One-way or two-way ANOVA was used for comparisons among multiple independent samples, with *P* < 0.05 considered statistically significant.

### Supplementary Information

**Additional file 1: Figure S1**. Biocompatibility of nanoscavengers. **Figure S2**. Survival rate of mice intrathecally poisoned by MP. **Figure S3**. GFAP-positive astrocytes were observed under a fluorescence microscope in the ctrl group and the 7, 14, and 28 d groups after MP poisoning. **Figure S4**. Latency to find the platform of MP-challenged mice. **Figure S5**. Latency to find the platform of MP-challenged mice treated with different antidotes.

## Data Availability

Not applicable.

## Abbreviations

OP: Organophosphate
BBB: Blood–brain barrier
AChE: Acetylcholinesterase
ACh: Acetylcholine
CNS: Central nervous system
E-Lipo: Erythrocyte-liposome
rOPH: Recombinant organophosphorus hydrolase
MP: Methyl paraoxon
IL-6: Interleukin-6
IL-1β: Interleukin-1β
TNF-α: Tumour necrosis factor-α
GFAP: Glial fibrillary acidic protein
FT-IR: Fourier transform infrared
XRD: *X*-Ray diffraction
DSC: Differential scanning calorimetry
PDI: Polydispersity index
DLS: Dynamic light scattering
TEM: Transmission electron microscope
HE: Haematoxylin–eosin
BMECs: Brain microvascular endothelial cells
ROS: Reactive oxygen species
SOD: Superoxide dismutase
GSH: Glutathione
PBS: Phosphate buffered saline
CA: Cornu ammonis
DG: Dentate gyrus
