# Supplementary material for: Brain-targeted nanoreactors prevent the development of organophosphate-induced delayed neurological damage
Source: J Nanobiotechnology. 2023 Aug 7;21:256. doi: 10.1186/s12951-023-02039-2 (PMC10405429; doi:10.1186/s12951-023-02039-2)
Supplement: Supplementary file 1 — Additional file 1: Figure S1. Biocompatibility of nanoscavengers. Figure S2. Survival rate of mice intrathecally poisoned by MP. Figure S3. GFAP-positive astrocytes were observed under a fluorescence microscope in the ctrl group and the 7, 14, and 28 d groups after MP poisoning. Figure S4. Latency to find the platform of MP-challenged mice. Figure S5. Latency to find the platform of MP-challenged mice treated with different antidotes. [file 12951_2023_2039_MOESM1_ESM.docx]

## Additional file Information

**Brain-Targeted Nanoreactors Prevent the Development of Organophosphate-Induced Delayed Neurological Damage**

Shuaijun Zou, ^1,†^ Qianqian Wang, ^1,†^ Qian He, ^2,†^ Guoyan Liu, ^1^ Juxingsi Song, ^1^ Jie Li, ^1^ Fan Wang, ^1^ Yichao Huang, ^1^ Yanan Hu, ^1^ Dayuan Zhou, ^1^ Yongfei Lv, ^1^ Yuanjie Zhu, ^3,*^, Beilei Wang, ^1, *^ Liming Zhang ^1,*^

^1^Department of Marine Biomedicine and Polar Medicine, Naval Special Medical Center, Naval Medical University, Shanghai 200433, China

^2^The Third Affiliated Hospital, Naval Medical University, Shanghai 200433, China

^3^Department of Marine Biological Injury and Dermatology, Naval Special Medical Center, Naval Medical University, Shanghai 200052, China

^*^Corresponding Authors: [13918338960@126.com](mailto:13918338960@126.com) (Y.Z.); [beileiwang@smmu.edu.cn](mailto:beileiwang@smmu.edu.cn) (B.W.); [lmzhang@smmu.edu.cn](mailto:lmzhang@smmu.edu.cn) (L.Z.), 021-81871129.

^†^ These authors contributed equally to this work.

# Additional file Results


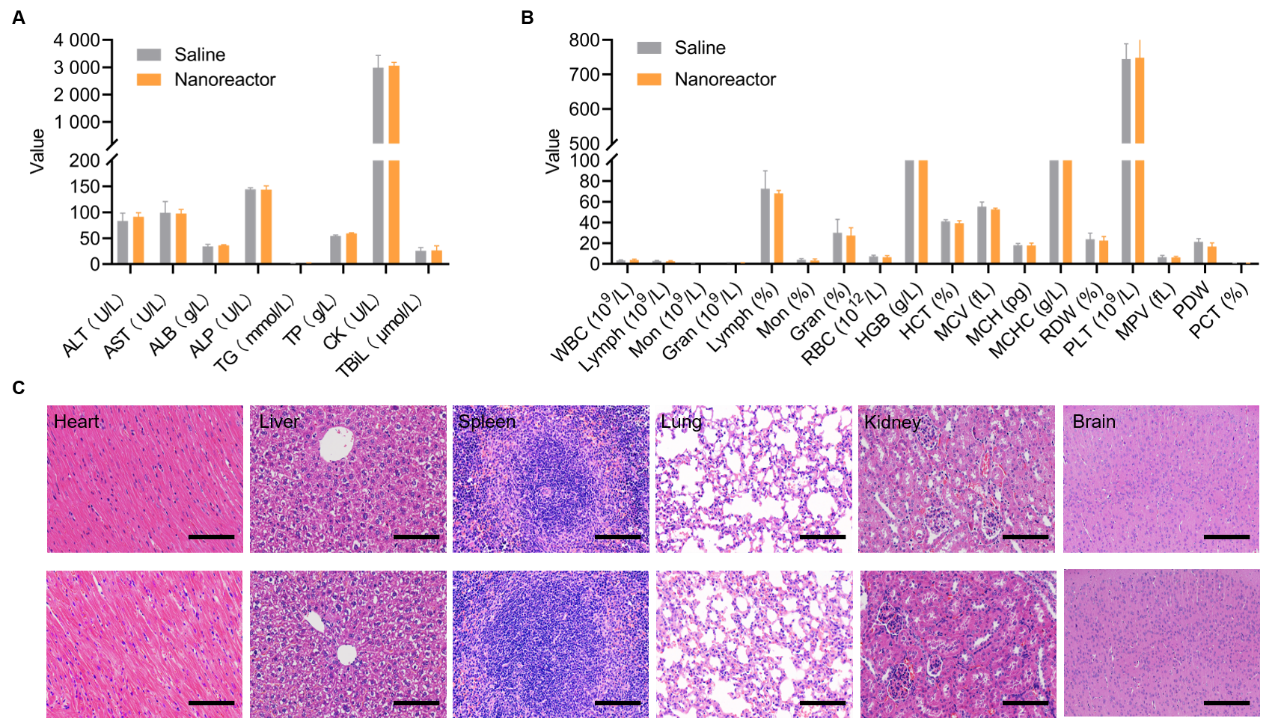


**Figure S1. Biocompatibility of nanoreactors. (A)** Blood biochemistry and **(B)** routine blood examination of mice with different treatments every other day for a week. **(C)** Histopathological examination of the major organs (heart, liver, spleen, lungs, kidneys and brain) via H&E staining. Scale bar = 50 μm. Data are shown as the means ± standard error (SE). n = 6. (Abbreviations: WBC: white blood cell; Lymph: lymphocyte; Mon: monocyte; Gran: granulocyte; RBC: red blood cell; HGB: haemoglobin; HCT: haematocrit; MCV: mean corpuscular volume; MCH: mean corpuscular haemoglobin; MCHC: mean corpuscular haemoglobin concentration; RDW: red cell distribution width; PLT: platelet; MPV: mean platelet volume; PDW: platelet distribution width; PCT: plateletcrit; ALT: alanine aminotransferase; AST: aspartate aminotransferase; ALB: albumin; ALP: alkaline phosphatase; TG: triacylglycerol; TP: total protein; CK: creatine kinase; TBiL: total bilirubin)


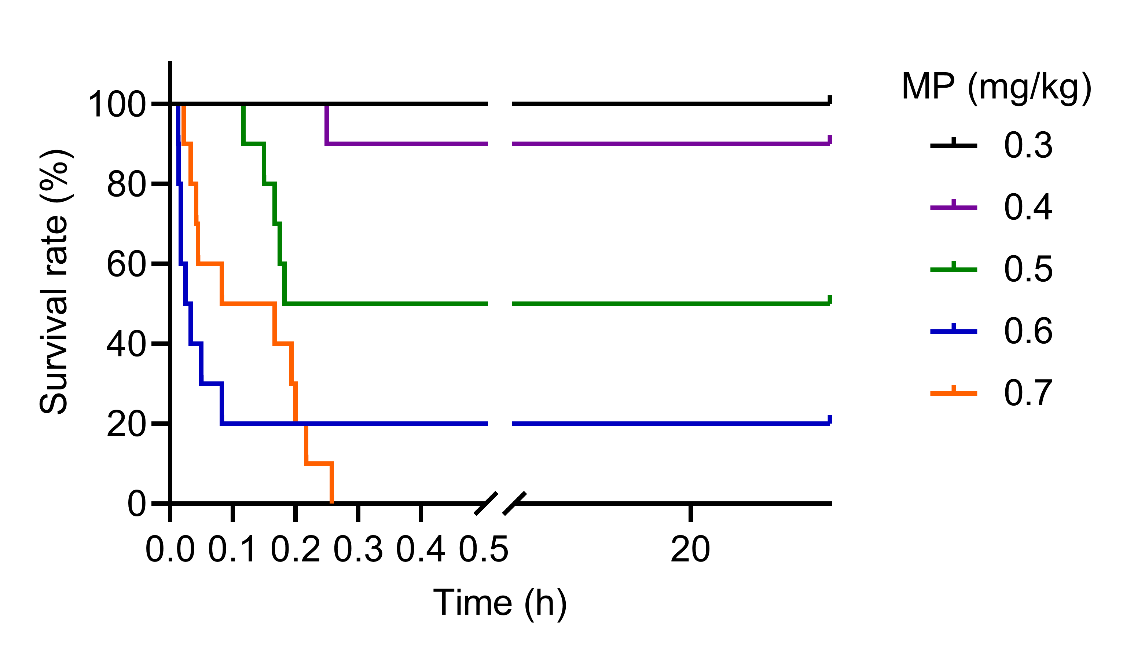


**Figure S2. Survival rate of mice intrathecally poisoned by MP at gradient concentrations.** n = 6


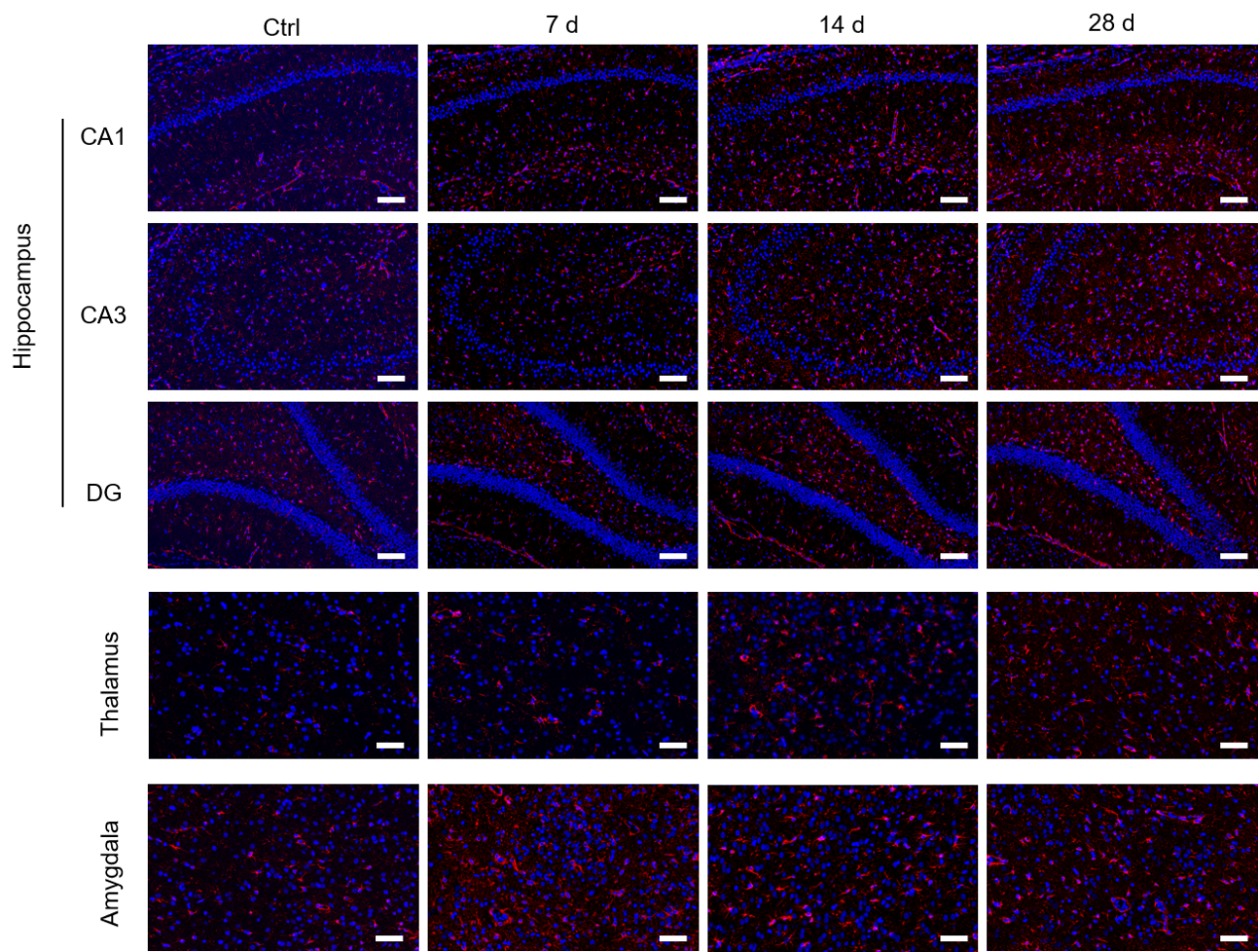


**Figure S3. GFAP-positive astrocytes observed under fluorescent microscope of the Ctrl group and the 7, 14, 28 d groups after MP poisoning.** n = 3


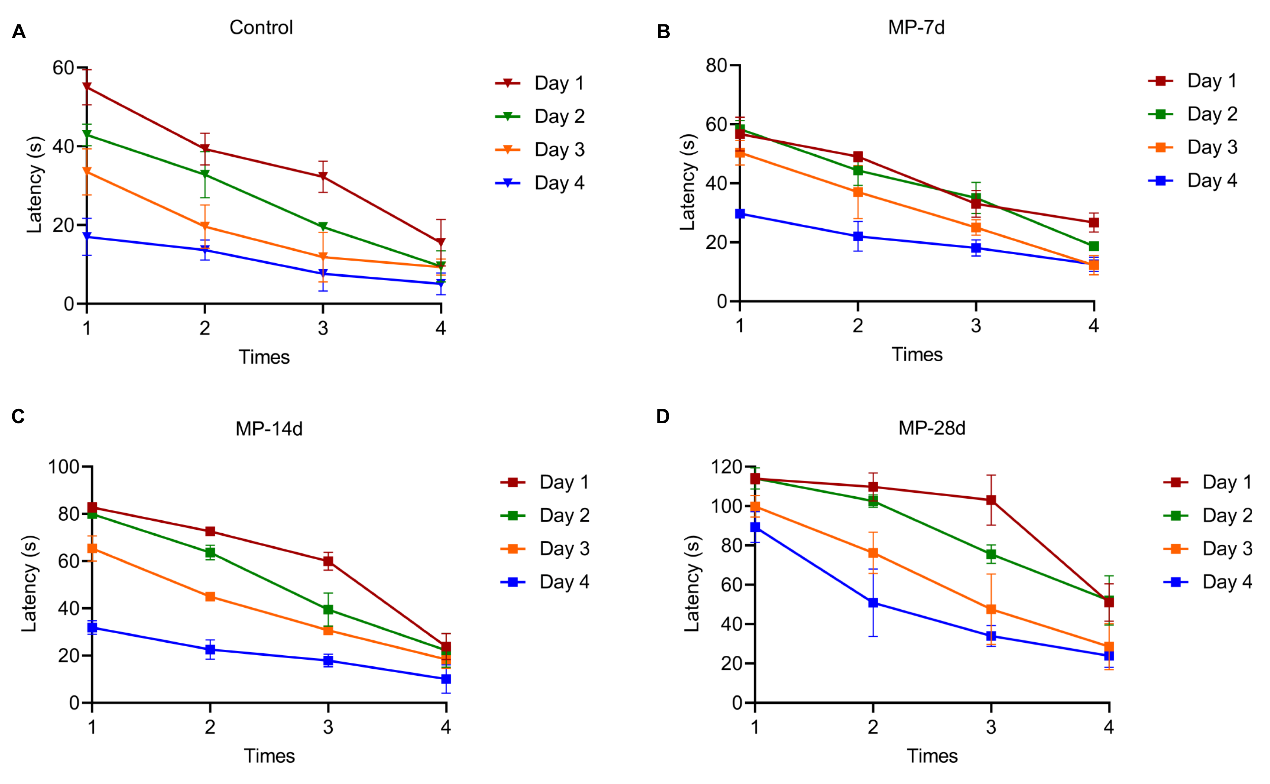


**Figure S4. Latency to find platform of MP-challenged mice.** Escape latency during four training days of **(A)** control mice, **(B)** MP-challenged mice at 7 d, **(C)** MP-challenged mice at 14 d, and **(D)** MP-challenged mice at 28 d. n = 3


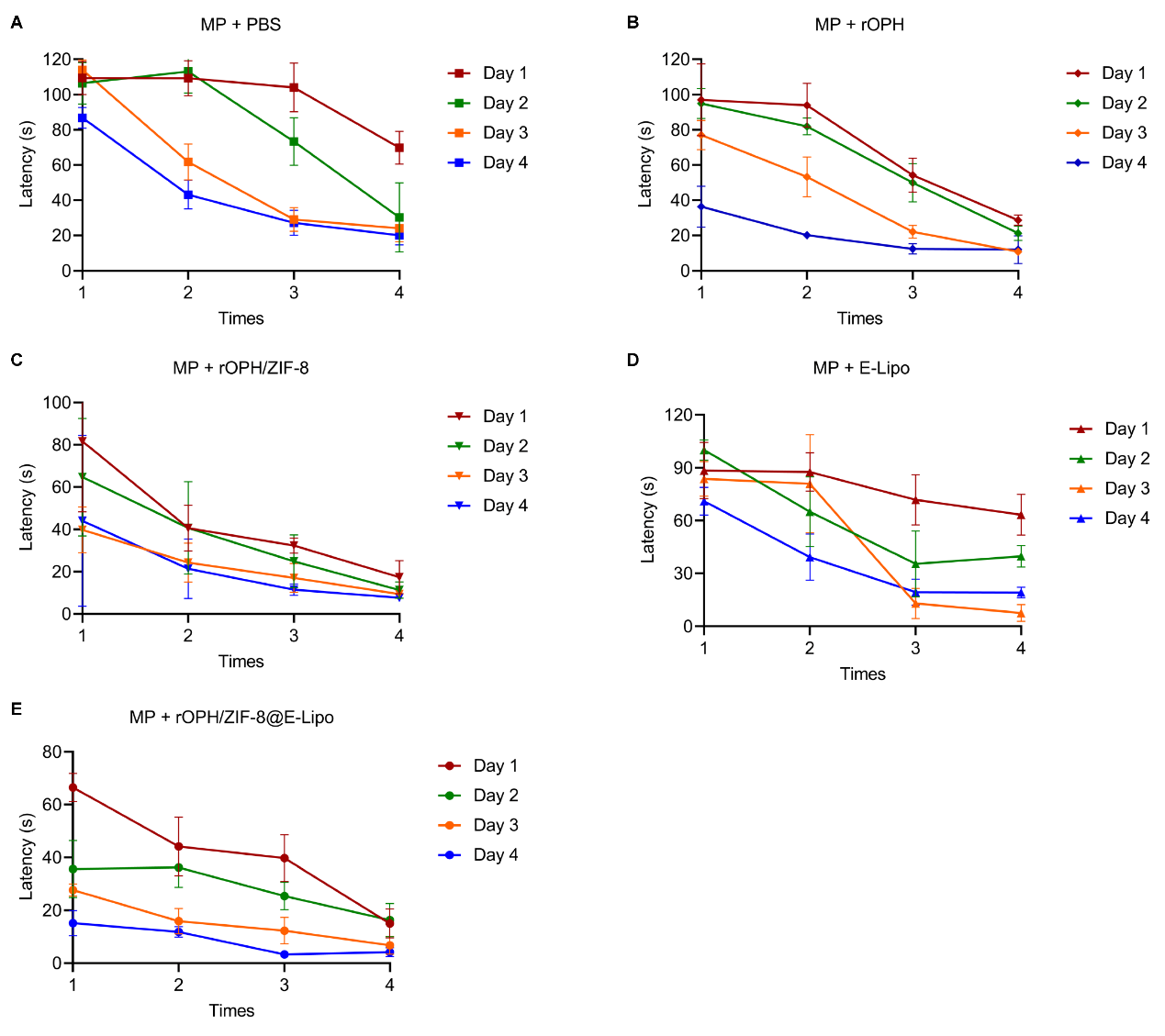


**Figure S5. Latency to find platform of MP-challenged mice with different** **antidotes (A)** PBS, **(B)** rOPH, **(C)** rOPH/ZIF-8, **(D)** E-Lipo, and **(E)** rOPH/ZIF-8@E-Lipo were injected to examine the preventive effects on cognitive deficits in terms of latency to find platform during four training days.
